# Primary IgA and IgG subclass deficiency in a 17-year-old Pakistani girl: a case report

**DOI:** 10.4076/1757-1626-2-6785

**Published:** 2009-08-10

**Authors:** Taimur Saleem, Madiha Rabbani, Bushra Jamil

**Affiliations:** 1 Medical College, The Aga Khan UniversityStadium Road, Karachi, 74800Pakistan; 2 Department of Medicine, The Aga Khan UniversityStadium Road, Karachi, 74800Pakistan

## Abstract

Primary immunodeficiency disorders pose a diagnostic dilemma for physicians in the developing countries such as Pakistan because of lack of adequate diagnostic facilities. We present here the case of a 17-year-old girl who had a history of recurrent respiratory tract infections since childhood and had been treated with anti-tuberculous medications thrice; for a total of 24 months. She had also received multiple courses of antibiotics. Her initial presentation to our hospital was with acute bronchopneumonia. Her past medical history of recurrent infections also alerted the treating physician to the possibility of bronchiectasis secondary to a variety of underlying potential pathologies such as post-infection, immunodeficiency syndromes or ciliary dyskinesia disorders. Cystic fibrosis was also an important consideration. Direct enquiry revealed that there was no history of consanguineous marriage in her parents. Her sweat chloride test was within normal range (<40 mmol/L). Blood analysis was performed which showed IgA, IgG2 and IgG4 deficiency. She has been following up at our hospital for the past few years. In that course of time, she has had multiple episodes of pneumonia, gastroenteritis and maxillary sinusitis. She was successfully treated with intravenous immunoglobulins on four occasions when she presented with systemic crisis secondary to severe systemic infection. She also developed biopsy proven intermediate grade non-Hodgkin’s lymphoma five years after the diagnosis of immunoglobulin deficiency was first made. This appeared to be a complication of her immunodeficient state. She has been receiving chemotherapy for the lymphoma. Physicians should be cognizant of the morbidity that primary immunodeficiency syndromes such as immunoglobulin deficiency can have in the form of multiple infections and increased risk of malignancies as seen in our patient.

## Introduction

Primary immunodeficiency disorders pose a diagnostic dilemma for physicians in the developing countries because of lack of adequate diagnostic facilities. The exact prevalence of these disorders in the developing countries is not well known owing to lack of epidemiological data and national registries. In Pakistan, there is no national registry for such disorders. In a single center study done in Egypt including a total of 64 patients, antibody deficiencies were identified in 35.9% of the patients while combined T and B cell immunodeficiencies were observed in 29.7% of the patients [1].

We present here the case of a 17-year-old girl who had a history of recurrent respiratory tract infections since childhood.

## Case presentation

A 17-year-old Pakistani girl presented to our hospital with the complaints of productive cough, vomiting and high grade fever for one week. A diagnosis of acute bronchopneumonia was made on the basis of physical examination (tachypnea, basilar pulmonary crackles, fever) and postero-anterior view (PA) chest X-ray (right apical cavitation). She was admitted to the hospital and treated with intravenous antibiotics. Her sputum cultures grew *Pseudomonas aeruginosa* and her antibiotics were modified accordingly.

Past medical history of the patient was significant for recurrent respiratory tract infections since childhood; many of these episodes were associated with otitis media without perforation of the tympanic membrane. She had visited multiple doctors in the past few years and had been treated for tuberculosis up to three times in the past for a total of twenty four months in addition to receiving multiple courses of antibiotics. Her sputum smears and cultures for acid fast bacilli had not been positive. Her past history was negative for signs and symptoms of malabsorption, recurrent cutaneous infections or regular nasal drip. She had a history of primary amenorrhea at the time of initial presentation to us. She weighed 41 kg and her body mass index was 19.5 kg/m^2^ at that time.

After an uneventful discharge from the hospital for the bronchopneumonia, the patient was followed up on an out-patient basis for further workup. In view of the history of recurrent infections, the possibility of bronchiectasis secondary to a variety of underlying pathologies such as post-infection, immunodeficiency syndromes or ciliary dyskinesia disorders was considered. Cystic fibrosis was also an important consideration. There was no history of consanguineous marriage in her parents. Computed tomography scan obtained at that time didn’t show features of bronchiectasis. Her sweat chloride test was done as part of the workup. Chemical analysis of a 58 mg sweat sample from the patient showed a result of 22 mmol/L. Her blood analysis for immunoglobulins were performed next, showing a deficiency of IgA, IgG subclass 2 and 4 while her IgE and IgM levels were all normal.

Within the next two years, she was readmitted multiple times for severe gastroenteritis, bronchopneumonia and maxillary sinusitis. In addition to several courses of intravenous and oral antibiotics, she also received intravenous immunoglobulins (IVIG) on four separate occasions to help her cope with crisis secondary to severe systemic infections. She showed a successful resolution of the crisis after administration of intravenous immunoglobulins. The possibility of regular monthly administration of IVIG was discussed with the patient but not opted for due to financial constraints.

About five years after the initial diagnosis of primary immunoglobulin deficiency was made, she presented with localized cervical lymphadenopathy and a month’s history of fever. Her laboratory tests showed anemia (hemoglobin = 9.4 g/dl), leukocytosis (total leukocyte count = 14.7 × 10^9^/L), thrombocytosis (platelets=441 × 10^9^/L) and a Lactate Dehydrogenase (LDH) of 404 IU/L. In view of her immunodeficiency, we immediately biopsied the cervical lymph nodes. Histopathological examination of the lymph nodes showed scattered cells with vesicular nuclei, occasionally prominent nucleoli and mitosis in the background of histiocytes, plasma cells and lymphocytes. Based on positivity of LCA, CD 20, CD 3 and CD 30 along with a proliferative index of 30-40, a provisional diagnosis of intermediate grade non-Hodgkin’s lymphoma was made. Bone marrow biopsy confirmed these findings.

A complete radiological work-up was done using CT with contrast. It showed no mediastinal lymphadenopathy, multiple enlarged lymph nodes in the neck at levels 1, 2, 3 and 4 bilaterally along with left supraclavicular lymph nodes, bilateral enhancing axillary lymph nodes, hepatosplenomegaly, multiple large enhancing notes in peri-pancreatic, aorto-caval, celiac axis, para-aortic and mesenteric locations. She is currently receiving chemotherapy for intermediate grade non-Hodgkin’s lymphoma.

Her last chest X-ray showed development of fibrotic changes in right upper, middle and lower lung zones as well as bronchiectatic changes in the left basilar region. This most likely occurred in association with the multiple respiratory infections the patient has had in the past.

## Discussion

Recurrent pyogenic infections are the leading clinical manifestation in patients with antibody deficiencies [1-3]. A study done in Germany showed the presence of IgG subclass deficiency in patients with chronic rhinosinusitis not responsive to antibiotic therapy [4]. Our patient had a history of recurrent respiratory infections since childhood and had received multiple courses of antibiotics in the past. She had also received up to three courses of anti-tuberculous treatment without her sputum smears or cultures ever being positive. Although antituberculous medications are associated with side effects such as hepatotoxicity and color blindness, this was probably initiated by her previous physicians on the basis of high prevalence of tuberculosis in the region as well as her clinical symptomatology without investigation of her immunoglobulin status.

Her past medical history raised the suspicion for an immunodeficiency disorder when she presented to us and laboratory testing confirmed the diagnosis. We tested her for cystic fibrosis using the sweat chloride test since it is a cost-effective and useful test to rule out the disorder. We then proceeded to check her immunoglobulin levels (total, class and subclass). Although not performed, another useful step in the diagnostic work up of this patient would have been the evaluation of her antibody response to *Haemophilus influenzae* and pneumococcal vaccines.

IgG subclass deficiencies are often under-diagnosed entities because of the often unaffected levels of total immunoglobulin isotypes. Total immunoglobulin levels were normal in our case. IgG subclass deficiency therefore requires a measurement of all four IgG subtypes for an accurate diagnosis as was the case in our patient [3]. The most common subclass deficiency is IgG2. This may be accompanied by decreased IgG4 with or without decreased IgA levels. The IgG2-subclass deficiency is associated with a reduced immune response to polysaccharide antigens [3]. This explains the propensity for infections with encapsulated organisms since polysaccharides form the main constituent of the capsule for these organisms.

Our patient had developed bronchiectatic changes in her left lung as a result of multiple respiratory tract infections while her right lung also showed extensive fibrotic changes. Acute-on-chronic infections represent the most common pathway for respiratory dysfunction in patients with immunodeficiency disorders [5]. In a study from Canada, development of bronchiectatic changes was seen in patients with antibody deficiency disorders particularly in right middle and lower lobes [6]. This will translate into significant morbidity in the long term for our patient. It has therefore been recommended that greater awareness regarding respiratory complications of such disorders be generated amongst the treating physicians so that this aspect of care is not overlooked in the treatment of such patients [5].

The possibility of tuberculosis associated bronchiectasis in our patient can’t be definitively ruled out. We can generally attribute the bronchiectatic pattern to multiple episodes of previous sinopulmonary infections. Our patient showed cystic bronchiectatic changes in the basilar areas. *Mycobacterium tuberculosis* has been reported to cause patterns of both cystic as well as cylindrical bronchiectasis on CT scan [7]. Non-fungal and non-tuberculous infections generally cause bronchiectasis in the lower lower lobes, the right middle lobe and the lingual. *M. tuberculosis* infection on other hand classically causes bronchiectatic changes in the upper lobes [8].

Procurement of IVIG therapy may also represent a potential hurdle in the long term treatment of these patients in developing countries. Studies have shown the useful role of regular IVIG therapy for prophylaxis against infections [9,10]. However, in developing countries, regular therapy is difficult because of issues such as cost, brief hospital admission required for administration of immunoglobulins, patient compliance with scheduling and possibility of contamination of the product given [10].

Although long term IVIG therapy is generally safe, it can cause adverse effects in some patients. The risks should therefore be weighed against the benefits and this merits a comprehensive discussion between the physician and the patient [10]. In a study including 45 immunodeficient patients in Iran who were studied during a 36 month period, the rate of adverse effects was 5.2% across a total of 955 infusions [11]. Adverse effects of IVIG therapy include rash, chills, flushing, fever, nausea, severe headache, joint pains, dyspnea, diarrhea, tachycardia and anaphylactic reactions [11,12]. More severe late adverse effects can include acute renal failure, thromboembolic events, aseptic meningitis, neutropenia, cutaneous reactions and autoimmune hemolytic anemia [12]. It has been recommended that patients on long term IVIG therapy should be monitored by a physician who is familiar with these risks and adverse effects [11].

## Conclusion

We want to highlight the following important issues using this case report. Primary immunodeficiency disorders impose significant morbidity on patients as was seen in our patient. This occurs in the form of multiple infections, increased risk of malignancies and various treatments. Our patient developed recurrent respiratory tract infections, recurrent gastroenteritis, bronchiectasis and intermediate grade non-Hogdkin’s lymphoma. She had received multiple courses of antibiotics and antituberculous therapy.

Antibody deficiencies may not be rare in children in the developing countries but they are certainly rarely reported in literature from these regions because of their high potential for underdiagnosis. This is primarily because of the lack of availability of adequate facilities to diagnose these disorders. We were not able to find any case report from our region using MEDLINE search on this subject. There is a lack of training and education of the physicians regarding these disorders. A high index of suspicion is needed on the part of physicians to timely diagnose these disorders so that appropriate treatments can be offered and instituted. These patients may then be referred to more specialized centers.

Highly vigilant outlook is needed on the part of doctors for the development of malignancies and bronchiectasis in these patients. Also, establishment of national registries in developing countries for these immunodeficiency disorders is an important future step [1].

## Abbreviations

IVIG: Intravenous immunoglobulins
LDH: lactate dehydrogenase
PA: posteroanterior
